# Perception, attitude, practice and barriers towards medical research among undergraduate students

**DOI:** 10.1186/s12909-020-02104-6

**Published:** 2020-06-17

**Authors:** Dina El Achi, Lara Al Hakim, Maha Makki, Mona Mokaddem, Pamela Abi Khalil, Bilal R. Kaafarani, Hani Tamim

**Affiliations:** 1 Department of Nutrition and Dietetics, American University of Beirut, Beirut, 1107-2020 Lebanon; 2 Clinical Research Institute, Faculty of Medicine, American University of Beirut, Beirut, 1107-2020 Lebanon; 3 Department of Biology, American University of Beirut, Beirut, 1107-2020 Lebanon; 4 Department of Chemistry, American University of Beirut, Beirut, 1107-2020 Lebanon; 5 Department of Internal Medicine, American University of Beirut Medical Center, Beirut, 1107-2020 Lebanon

**Keywords:** Medical research, Undergraduate students, Undergraduate medical research, Medical Research Volunteer Program (MRVP), Attitude, Barriers, Perception

## Abstract

**Background:**

Early exposure to medical research, specifically at the undergraduate level, contributes in building a solid medical education for students. In 2014, the Medical Research Volunteer Program (MRVP) was established at the American University of Beirut (AUB). This program provides undergraduate students with the opportunity to be involved in ongoing medical research projects, on a voluntary basis. Little is known about undergraduates’ outlook on medical research and the challenges they face. The aim of this study was to assess, among AUB undergraduate students, the perception, attitude, practice and barriers towards medical research, as well as to identify factors affecting them, such as background characteristics and research involvement.

**Methods:**

A cross sectional study was carried out at AUB, where undergraduate students enrolled in specific chosen classes were surveyed in spring 2019 via paper based questionnaires. Close-ended questions assessed students’ attitude, perception, practice and barriers towards medical research using a 5-point likert scale. Moreover, demographic characteristics and research involvement information were collected.

**Results:**

Five hundred and twenty three students were surveyed. About half of the students were premedical students (51.5%), and only 43.6% of them were aware of the medical research program at their university. The average attitude, perception, practice and barriers scores were found to be 3.58, 4.35, 3.58 and 2.60, respectively (on a scale from 1 to 5). Students found the lack of mentoring and guidance to be the main barrier in conducting medical research.

**Conclusion:**

Our findings reveal that students express interest towards medical research and recognize its importance. A medical research program at the undergraduate level is indispensable in guiding students in their future career endeavors. Therefore, research programs should be constantly monitored to assure a research-oriented environment within institutions.

## Background

Health sciences research and new scientific innovations are currently guiding clinical practice and becoming an essential part of medical education [1]. By incorporating research in their educational systems, medical schools promote a pool of researchers and allow students to acknowledge their career prospects. For instance, a medical school in Sudan modified its curricula to train students in research [2]. Studies suggest that early exposure to research, specifically at the undergraduate level, can be considered as a natural catalyst in building a solid medical education for students [2].

At the graduate level, there have been several studies assessing students’ perception and attitude towards medical research. In fact, a study by Funston et al. [3] revealed that, among 1625 responses collected from 38 countries, less than half of medical students believed that their medical schools provided opportunities to be involved in mentored research. Another study by Vodopivec et al. [4] assessed medical students’ knowledge and attitude towards scientific research in Croatia. This study revealed that students expressed a positive attitude towards research. Other studies have shown that some students have inadequate knowledge in research and are unaware of its importance which results in a compromised exposure to research training [2]. However, there are only a few studies [5] assessing the nature of the barriers faced by graduate or undergraduate students while conducting medical research.

As for undergraduate students’ involvement in medical research, very little is known about their perception, attitude, practice and barriers towards medical research. In Lebanon, the Medical Research Volunteer Program (MRVP) [6] was established in 2014 to help undergraduate students be involved in medical research and to help students overcome the challenges they face. This program provides undergraduate students at the American University of Beirut (AUB) with active research experience early on in their careers, by matching them with researchers at the American University of Beirut Medical Center (AUBMC). Matched students become part of a research team and assist in different research-related tasks, on a volunteer basis. The MRVP has become a platform for AUB undergraduate students interested in gaining a deeper understanding of the medical research process, and gaining hands on experience with prominent physicians.

In this study, we aimed to assess the perception, attitude, practice and barriers towards medical research among AUB undergraduate students and to identify factors affecting them, such as demographic characteristics, educational background, and research involvement.

## Methods

### Study design and setting

A cross sectional study was carried out between January and February 2019 at the AUB. Undergraduate students in four faculties were invited to participate in this study. Eligible students included those aged 18 and above in sophomore, junior and senior levels during spring semester of the 2018–2019 academic year. Freshman students, graduate students, majorless students, students under 18 years of age, and students who did not agree to participate in the study were excluded from this study.

### Selection of participants

Students who participated in this study were those enrolled in one of the four following faculties: Faculty of Arts and Sciences (FAS), Faculty of Agricultural and Food Sciences (FAFS), Faculty of Health Sciences (FHS) and the Rafic Hariri School of Nursing (HSON). In 2019, these faculties included 3038, 396, 251, and 166 undergraduate students, respectively. A list of courses from these four faculties was generated to represent the students’ different fields and academic levels (sophomore, junior and senior). Accordingly, biology, chemistry, physics, nutrition, environmental health, medical laboratory sciences, medical audiology sciences, medical imaging sciences, and nursing courses were chosen from each level to ensure capturing the majority of students at different levels in these majors. These courses included a number of students proportional to the total number of students in each faculty. Moreover, students enrolled in these classes are most prone to conduct medical research, because many of them are pre-medical students. After acquiring the approvals from the deans of these four faculties and those of the professors of the chosen classes, the members of the research team attended the chosen classes at a pre-determined time. After explaining the objective and process of the study to all students, the research team distributed the questionnaire only to those who agreed to participate. Sample size calculation was based on convenience rather than statistical grounds as the main objective of this study was descriptive in nature. Accordingly, the sample was randomly selected from AUB undergraduate pool in a stratified manner based on faculty, field, and year of study, and this included 523 students.

### Data collection

The questionnaire used in this study was developed using the investigators’ previous experience in this field, as well as previously published work that is relevant to this paper. More specifically, Vodopivec et al. [4] used a questionnaire to assess the attitude of first year medical students towards research in Croatia.

The final draft of the questionnaire used in this study included four sections:

1. The first part covered demographic information and educational status of the participants such as age, gender, class standing, major, and self-reported Grade Percentage Average (GPA).
2. The second part examined the participants’ familiarity with MRVP, how students learned about MRVP, their participation in MRVP, and the duration of their participation.
3. The third part examined the participants’ involvement in medical research such as the field of medical research conducted and previous or current publications or presentations.
4. The fourth part was divided into four subsections addressing the following:
  - Participants’ attitudes towards medical research, such as the enjoyment, the excitement, the complexity, and the value of medical research.
  - Participants’ perceptions towards medical research, such as the role of medical research in the enhancement of knowledge and career prospects.
  - Participants’ medical research practice assessed by their willingness to take part in any research related task and spend more than 2 months working on a medical research project.
  - Participants’ barriers towards medical research, such as lack of time, lack of mentorship, and lack of exposure.

Attitude, perception, practice and barriers towards medical research were assessed using Likert scale, ranging from 1 to 5 (1 = strongly disagree; 2 = disagree, 3 = neither agree nor disagree, 4 = agree, 5 = strongly agree). We categorized students’ responses to questions concerning attitude, perception, practice and barriers into positive and negative answers. For statements favoring medical research (such as “Medical research is enjoyable”), a positive answer corresponded to strongly agreeing, agreeing or neither agreeing nor disagreeing, while a negative answer corresponded to strongly disagreeing or disagreeing. The opposite was true for statements that did not favor medical research (such as “Medical research is time consuming”).

In addition, an open-ended question was added to capture any further comments, concerns, or feedback that the students had.

The developed questionnaire was pilot tested among 16 undergraduate students to examine its clarity and reliability. Their feedback was mainly related to the structure of the questionnaire, and the ambiguity of some of the original questions. Accordingly, these comments were taken into consideration and the questionnaire was modified to attain the final draft distributed to the student sample included in the study. The results of these participants were not included in the final data analyzed for this study.

The co-investigators attended the last 15 min of the chosen classes of the first week of the Spring semester, and explained to the students the objectives of the study. The students were informed that their participation is completely voluntary and that there are no direct benefits stemming from it. Members of the research team also informed the students that they can skip a question if they do not wish to answer it, address any question to one of the members of the research team, and stop their participation at any point. Students who have already filled out a questionnaire from a previous class were asked not to fill it out again. The co-investigators then administered the 15-min questionnaire to the students who had wished to participate and left the class for maximum privacy and anonymity.

### Statistical analyses

Data was manually entered into excel and then transferred to the Statistical Package for Social Sciences (SPSS) software version 24, which was used for data analyses. Participants who did not fill out all the questions in the survey were removed from the sample. Participants who answered both yes and no on close-ended questions were also removed from the sample. Categorical variables were summarized using numbers and percentages while continuous variables were summarized by means ± standard deviation (SD). A one-way analysis of variance (ANOVA) was used to compare continuous variables with more than two categories, and an independent t-test was used for those with two categories. Bivariate analysis for the perception, attitude, practice, and barriers, demographic characteristics and research involvement were assessed. A *p*-value ≤0.05 was considered statistically significant.

## Results

### Demographic characteristics

A total of 523 students participated in the survey. The overall response rate was 40.0% (523 out of 1306 registered in the courses that were surveyed). Response rates were 33.3% (*N* = 174) from sophomores, 27.9% (*N* = 146) from juniors and 38.8% (*N* = 203) from seniors. Three hundred and fifty five (68%) students were female and the majority of students (93.9%) were aged between 18 and 21. About half of the students were premedical students (*N* = 267, 51.1%). The majority of students were biology majors (*N* = 161, 31.0%) and the minority were math and physics students (*N* = 19, 3.7%). As for research involvement, slightly more than half of the students (*N* = 295, 56.4%) were unaware of the MRVP at AUB. Moreover, 91.9% (*N* = 477) of the students did not have previous or current research experience excluding research under the MRVP. About half the students with previous or current research experience attained research publications (*N* = 19, 46.3%) (Table 1).

**Table 1:** Demographic characteristics and research involvement

Characteristics		Number (percent) ***N*** = 523
**Age**	18–21	491 (93.9)
	22–25	28 (5.4)
	25+	4 (0.8)
**Sex**	Male	167 (32.0)
	Female	355 (68.0)
**Class**	Sophomore	174 (33.3)
	Junior	146 (27.9)
	Senior	203 (38.8)
**Credits,** mean ± SD		30 ± 4.42
**Major**	Agriculture/Nutrition	118 (22.7)
	Math/physics	19 (3.7)
	Biology	161 (31.0)
	Chemistry	65 (12.5)
	Environmental heath/Nursing	133 (25.6)
	Other^a^	23 (4.4)
**Premedical**		267 (51.1)
**GPA**	< 70%	14 (2.7)
	70–78%	176 (34.2)
	79–85%	146 (28.3)
	86–90%	121 (23.5)
	> 90%	58 (11.3)
**MRVP familiarity**		228 (43.6)
**MRVP participation duration (months),** mean ± SD		5.56 ± 4.68
**Other previous/current research experience**		42 (8.1)
**Other previous/current research experience duration (months),** mean ± SD		5.98 ± 4.90
**Publications**		19 (46.3)
**Presentations**		19 (47.5)

^a^The “Other” section includes the following majors: psychology, political sciences, education, mechanical engineering, electrical and computer engineering, computer and communications engineering and chemical engineering

### Perception

The questionnaire included 3 items that addressed the perception of students towards medical research. It was found that the majority of students had a positive perception for the following three items, medical research promoting critical thinking, enhancing one’s career prospect, and enhancing knowledge (99.4, 98.8, and 99.4%, respectively) (Table 2). As for the score created out of these items, the average was found to be 4.35 ± 0.57. When stratified by different demographic characteristics and research involvement information, research showed a positive association between the perception scores and students’ Grade Percentage Average (GPA) (data not shown). As students’ GPA increased, they had a better perception towards medical research (4.15 ± 0.59 for GPA < 70% and 4.45 ± 0.76 for GPA > 90%), with no statistically significant difference (*p* = 0.38) (Table 2). Similarly, females, sophomores, and premedical students reported a more positive perception towards medical research as indicated by their scores (*p*-values of 0.13, 0.08, and 0.06, respectively).

**Table 2:** Positive perception responses among students, as well as overall perception score and those stratified by different students’ characteristics

Perception		Positive responses	
Enhances critical thinking		516 (99.4%)	
Enhances knowledge		515 (99.4%)	
Enhancing career prospect		513 (98.8%)	
Overall score, mean ± SD		4.35 ± 0.57	
**Stratified by students’ characteristics**		**Mean ± SD**	***P*-value**
**Sex**	Male	4.29 ± 0.67	0.13
	Female	4.38 ± 0.51	
**Age**	18–21	4.35 ± 0.57	0.54
	22–25	4.46 ± 0.53	
	25+	4.25 ± 0.50	
**Class standing**	Sophomore	4.43 ± 0.53	0.08
	Junior	4.29 ± 0.56	
	Senior	4.34 ± 0.60	
**GPA**	< 70%	4.15 ± 0.59	0.38
	70–78%	4.33 ± 0.58	
	79–85%	4.33 ± 0.49	
	86–90%	4.39 ± 0.55	
	> 90%	4.45 ± 0.76	
**Major**	Agriculture/Nutrition	4.40 ± 0.54	0.15
	Math/physics	4.30 ± 0.56	
	Biology	4.38 ± 0.57	
	Chemistry	4.19 ± 0.74	
	Environmental heath/Nursing	4.34 ± 0.52	
	Other^a^	4.50 ± 0.45	
**Premedical**	Yes	4.40 ± 0.59	0.06
	No	4.31 ± 0.55	
**MRVP participation**	Yes	4.49 ± 0.58	0.22
	No	4.38 ± 0.54	
**Other previous/current research experience**	Yes	4.42 ± 0.49	0.43
	No	4.35 ± 0.57	

^a^The “Other” section includes the following majors: psychology, political sciences, education, mechanical engineering, electrical and computer engineering, computer and communications engineering and chemical engineering

### Attitude

Student’s attitude was assessed using five factors. The majority of students had a positive attitude towards medical research in terms of medical research being valuable, exciting, enjoyable, complicated, and time-consuming (98.8, 97.7, 96.2, 91.3, and 76.1%, respectively) (Table 3). As for the score created out of these 5 items, the average was found to be 3.58 ± 0.48. Sophomores had a significantly higher attitude score compared to juniors and seniors (3.71 ± 0.47 vs. 3.54 ± 0.46 vs. 3.51 ± 0.48 with *p* < 0.0001). Premedical students also had a significantly higher attitude score compared to non-premedical students (3.67 ± 0.45 vs. 3.50 ± 0.49 with *p* < 0.0001) (Table 3). Females and mathematics/physics majors had a higher attitude score (*p*-values of 0.46 and 0.13, respectively). Research showed a positive association between the attitude scores and students’ GPA (3.49 ± 0.61 for GPA < 70% and 3.65 ± 0.48 for GPA > 90%), with no statistically significant difference (*p* = 0.72). In terms of research involvement, students who have participated in MRVP and those who took part in other research projects expressed a higher attitude (*p*-values of 0.34 and 0.39, respectively) (Table 3).

**Table 3:** Positive attitude responses among students, as well as overall attitude score and those stratified by different students’ characteristics

Attitude		Positive responses	
Valuable		512 (98.8%)	
Exciting		509 (97.7%)	
Enjoyable		501 (96.2%)	
Complicated		472 (91.3%)	
Time consuming		395 (76.1%)	
Overall score, mean ± SD		3.58 ± 0.48	
**Stratified by students’ characteristics**		**Mean ± SD**	***P*-value**
**Sex**	Male	3.56 ± 0.50	0.46
	Female	3.60 ± 0.46	
**Age**	18–21	3.59 ± 0.48	0.85
	22–25	3.57 ± 0.53	
	25+	3.45 ± 0.25	
**Class standing**	Sophomore	3.71 ± 0.47	< 0.0001
	Junior	3.54 ± 0.46	
	Senior	3.51 ± 0.48	
**GPA**	< 70%	3.49 ± 0.61	0.72
	70–78%	3.58 ± 0.49	
	79–85%	3.56 ± 0.42	
	86–90%	3.59 ± 0.50	
	> 90%	3.65 ± 0.48	
**Major**	Agriculture/Nutrition	3.53 ± 0.47	0.13
	Math/physics	3.78 ± 0.54	
	Biology	3.65 ± 0.43	
	Chemistry	3.58 ± 0.62	
	Environmental heath/Nursing	3.53 ± 0.47	
	Other^a^	3.61 ± 0.34	
**Premedical**	Yes	3.67 ± 0.45	< 0.0001
	No	3.50 ± 0.49	
**MRVP participation**	Yes	3.68 ± 0.46	0.34
	No	3.62 ± 0.40	
**Other previous/current research experience**	Yes	3.64 ± 0.52	0.39
	No	3.58 ± 0.47	

^a^The “Other” section includes the following majors: psychology, political sciences, education, mechanical engineering, electrical and computer engineering, computer and communications engineering and chemical engineering

### Practice

Medical research practice was assessed by students’ willingness to take part in any research related task, to spend more than 2 months on a research project, to take part in a project even if it does not lead to a publication and to devote the same time for medical research as their university studies. The majority of students were positive about these factors (92.15, 88.4, 87, and 68.3%, respectively) (Table 4). As for the score created out of these items, the average was found to be 3.57 ± 0.78. When stratified by demographic characteristics, premedical students had a significantly higher practice score than non-premedical students (3.71 ± 0.7 vs. 3.44 ± 0.84 with *p* < 0.0001). When stratified by research involvement, students who have participated in MRVP and those who are undergoing or have undergone medical research excluding MRVP, expressed more willingness as indicated by a statistically significant higher practice score (both *p*-values < 0.0001). Results also indicated a positive association between GPA and practice scores (3.48 ± 0.97 for GPA < 70% and 3.61 ± 0.73 for GPA > 90%), with no statistically significant difference (*p* = 0.48) (Table 4).

**Table 4:** Positive practice responses among students, as well as overall practice score and those stratified by different students’ characteristics

Practice		Positive responses	
Willing to take part in any research related task		477 (92.1%)	
Willing to spend > 2 months		458 (88.4%)	
Willing to take part in a medical research project even if it does not lead to a publication		450 (87.0%)	
Willing to devote the same time to medical research as their university studies		354 (68.3%)	
Overall score, mean ± SD		3.57 ± 0.78	
**Stratified by students’ characteristics**		**Mean ± SD**	***P*-value**
**Sex**	Male	3.56 ± 0.83	0.67
	Female	3.60 ± 0.76	
**Age**	18–21	3.58 ± 0.75	0.79
	22–25	3.60 ± 1.20	
	25+	3.31 ± 0.63	
**Class standing**	Sophomore	3.67 ± 0.74	0.15
	Junior	3.54 ± 0.72	
	Senior	3.52 ± 0.85	
**GPA**	< 70%	3.48 ± 0.97	0.48
	70–78%	3.56 ± 0.81	
	79–85%	3.50 ± 0.78	
	86–90%	3.68 ± 0.76	
	> 90%	3.61 ± 0.73	
**Major**	Agriculture/Nutrition	3.50 ± 0.79	0.21
	Math/physics	3.54 ± 0.91	
	Biology	3.71 ± 0.72	
	Chemistry	3.52 ± 0.87	
	Environmental heath/Nursing	3.52 ± 0.78	
	Other^a^	3.70 ± 0.83	
**Premedical**	Yes	3.71 ± 0.70	< 0.0001
	No	3.44 ± 0.84	
**MRVP participation**	Yes	3.96 ± 0.64	0.01
	No	3.67 ± 0.78	
**Other previous/current research experience**	Yes	3.90 ± 0.66	0.04
	No	3.55 ± 0.79	

^a^The “Other” section includes the following majors: psychology, political sciences, education, mechanical engineering, electrical and computer engineering, computer and communications engineering and chemical engineering

### Barriers

The questionnaire included 4 items that addressed students’ barriers towards medical research. A lack of allotted time for medical research was found to be the most predominant barrier (80.3%) followed by a lack of exposure and opportunities (79.9%), a lack of training and support (78.3%), and finally a lack of mentoring and guidance (76.6%) (Table 5). As for the score created out of these items, the average was found to be 2.60 ± 0.76. Males perceived significantly more challenges than females (2.75 ± 0.79 vs. 2.53 ± 0.74 with *p* = 0.002). Significant differences were found between the barriers score of students in different majors (*p* = 0.03). Students in agriculture/nutrition/food sciences and mathematics/physics categories perceived the least challenges (2.49 ± 0.75 and 2.42 ± 0.84, respectively), while students in the “others” category which includes engineering, political sciences, psychology and education perceived the most challenges (2.87 ± 0.72). In addition, premedical students perceived significantly more barriers than non-premedical students (2.67 ± 0.74 vs. 2.52 ± 0.78 with *p* = 0.04). Students who have participated in MRVP, are undergoing, or have undergone medical research excluding MRVP have a statistically significant higher barriers score (both *p*-values< 0.0001) (Table 5).

**Table 5:** Positive barriers responses among students, as well as overall barriers score and those stratified by different students’ characteristics

Barriers		Positive responses	
Lack of allotted time		403 (80.3%)	
Lack of exposure and opportunities		401 (79.9%)	
Lack of training and support		393 (78.3%)	
Lack of mentoring and guidance		384 (76.6%)	
Overall score, mean ± SD		2.60 ± 0.76	
**Stratified by students’ characteristics**		**Mean ± SD**	***P*-value**
**Sex**	Male	2.75 ± 0.79	0.002
	Female	2.53 ± 0.74	
**Age**	18–21	2.58 ± 0.75	0.2
	22–25	2.78 ± 0.86	
	25+	3.06 ± 0.88	
**Class standing**	Sophomore	2.60 ± 0.73	0.16
	Junior	2.50 ± 0.73	
	Senior	2.67 ± 0.80	
**GPA**	< 70%	2.65 ± 0.97	0.87
	70–78%	2.58 ± 0.75	
	79–85%	2.57 ± 0.78	
	86–90%	2.58 ± 0.74	
	> 90%	2.70 ± 0.79	
**Major**	Agriculture/Nutrition	2.49 ± 0.75	0.03
	Math/physics	2.42 ± 0.84	
	Biology	2.72 ± 0.75	
	Chemistry	2.65 ± 0.91	
	Environmental heath/Nursing	2.50 ± 0.68	
	Other^a^	2.87 ± 0.72	
**Premedical**	Yes	2.67 ± 0.74	0.04
	No	2.52 ± 0.78	
**MRVP participation**	Yes	3.27 ± 0.83	< 0.0001
	No	2.58 ± 0.72	
**Other previous/current research experience**	Yes	3.09 ± 0.89	< 0.0001
	No	2.55 ± 0.73	

^a^The “Other” section includes the following majors: psychology, political sciences, education, mechanical engineering, electrical and computer engineering, computer and communications engineering and chemical engineering

## Discussion

In this cross sectional study carried out at AUB to assess undergraduate students’ perception, attitude, practice and barriers towards medical research, we found an overall positive perception and attitude among study participants, as well as identified few barriers to getting involved in medical research at the undergraduate level.

Out of the total number of registered students in the selected classes in this study, a response rate was found to be 40%. Other studies [7, 8] assessing undergraduate students’ perception towards research generated higher response rates (100 and 74%, respectively). The response rate in our study was calculated on the basis of the number of students registered in each class rather than the total number of students having attended the class at the time. Our response rate could be explained by the fact that the data was collected during the first 2 weeks of the 2019 spring semester, with the first week being the drop and add period. We have selected this period not to disrupt the flow of classes at a later point in the semester.

Overall, students in our study expressed a positive perception, attitude and practice towards medical research as indicated by their scores, and a relatively low barriers score. Although, to the best of our knowledge, there are no reports addressing this question among undergraduate students, similar studies were conducted among medical students. Several of these studies revealed positive attitude towards medical research among medical students [5, 9, 10]. Although previous reports did not account for barriers scores, most revealed common barriers such as lack of time and lack of mentoring and guidance [5, 7, 11]. The similarity between our study and these studies can be attributed to the rise of several institutional programs at the undergraduate levels similar to the MRVP [11], and those at the graduate level targeting medical students aiming to improve the overall medical research experience [11].

### Gender

When stratified according to gender, our study revealed that females had a more positive perception, attitude and practice towards medical research, whereas males significantly perceived more barriers towards medical research. Carrying out a thorough literature review yielded few studies that assessed the above mentioned criteria in terms of gender. Contrary to our study, Khan et al. [12] and Noorleahi et al. [5] reported in their respective studies a higher attitude score among male medical students in Pakistan and Saudi Arabia, respectively. As for barriers, a study conducted by Funston et al. [3], reported opposite results compared to our results where females perceived more barriers towards medical research. These inconsistencies in findings could be attributed to societal, institutional, and personal factors. As for the societal factors, it could be due to the involvement of more females in male-oriented careers among the Lebanese culture. Regarding institutional support, we believe that with the implementation of the MRVP at AUB, students tend to feel more exposed and prepared to conduct medical research with a structured and opportunistic program. Finally, personal factors such as the stage of the educational journey (undergraduate versus medical students) and self-confidence might affect students’ barriers towards medical research as reported in a study by Burgoyne et al. [13].

### GPA and class standing

In our study, students with higher GPAs had a more positive perception, attitude and practice towards medical research A recent study by Ismail et al. [14] revealed a significant positive correlation between undergraduate students’ GPA and self-efficacy (*p*-value of 0.01). Students with a higher GPA tend to be more confident in their educational skills and capacities. They could therefore allow themselves to invest their capacities in medical research and perceive it positively. Students with higher GPAs might also perceive medical research as a catalyst for boosting their overall knowledge and thus possess a better perception towards medical research.

In terms of class standing, as students progressed in their class standing, their attitude became significantly less positive. In fact, sophomores had the most positive attitude towards medical research followed by juniors and seniors. Vujaklija et al. [15] reported that as students progressed in their medical school years, they expressed a significantly more positive attitude (*p*-value of 0.011) towards medical research with the introduction of a mandatory scientific methodology course. Our thought is that undergraduate students, contrary to medical students, are at the peak of their enthusiasm during their sophomore years and this enthusiasm tends to decline as they progress through their university years, where admission to the medical school becomes a priority with more focus on grades rather than research activities. Medical students on the other hand, progressively build interest in medical research as they progress through their medical student years [16].

### Premedical vs. non-premedical students

In this study, we have enrolled both premedical and non-premedical undergraduate students. For students wishing to apply for medical school according to the American curriculum (such as AUB), premedical undergraduate requirements include a pre-specified number of credits of biology, chemistry, physics, English and social sciences/humanities. These students must also earn a bachelor’s degree in any field, as long as they meet the above mentioned requirements [17]. Naturally, the premedical students form the pool of students who are potentially most interested in medical research. However, non-premedical students may also be interested in medical research. In fact, the MRVP program offers all students, regardless if premedical or not, the chance to be part of medical research at AUB. Moreover, the program has matched students from the two different pools on medical research programs since its inception. Therefore, we found it pivotal to include both premedical and non-premedical students in our sample.

Overall, premedical students had a more positive attitude, perception and willingness to conduct medical research as indicated by their higher scores. Although there was no study assessing these factors for premedical students, the results of a recent study by Pacifici et al. [18] relates to our results. Pacifici et al. revealed that premedical students, as compared to non-premedical students, use undergraduate research as a tool to help them get into medical school. Accordingly, we believe that premedical students, having paved their path towards medical school, view undergraduate medical research as an added value for their medical school applications. Thus, they are expected to express a more positive perception, attitude, and willingness towards medical research. Furthermore, our study also showed that premedical students perceived significantly more barriers in conducting medical research. Although our research did not yield any studies assessing premedical students’ barriers towards medical research, we found a recent study by Osman et al. [2] assessing medical students’ barriers towards medical research. This study revealed that 59.8% of medical students do not get involved in the medical research process because it is time consuming and they would rather focus on the demand of their institution’s curriculum. Similar to medical students, premedical undergraduate students also seem to have time constraints as their main barrier to conduct medical research. A potential solution could be getting involved in a medical research project that is of interest to them, rather than merely using it as a means to get into medical school. This way, students might feel more inclined to allocate medical research its proper time.

### MRVP participation/research involvement

Quantitative analysis of students’ attitude, perception and practice responses stratified by research involvement, elicited predominantly positive responses. Students who have participated in the MRVP expressed a more positive attitude and perception towards medical research. To our knowledge, such analysis only exists for medical students. A recent study by Houlden et al. [19] established that after the implementation of a research elective among medical students, there was a significant increase in the number of students who valued medical research’s role in developing critical thinking. Another study by Siemens et al. demonstrated that medical students with prior medical research experience tend to have a better attitude towards research [20]. We speculate that a solid medical research experience (as that offered by the MRVP) exposes undergraduate or medical students to the processes implicated in this type of research. This allows them to acknowledge its benefits such as its role in enhancing knowledge and critical thinking and thus result in a more positive medical research attitude, perception, and practice.

Among the whole sample, more than half (68.3%) of the students in our study were not willing to devote the same time to medical research as to their university studies, while others were unaware of the research activities and programs at their university.

After conducting a thorough literature search, we found several barriers impeding medical students from conducting medical research. Some might face ambivalence concerning a suitable balance between their clinical education and medical research [21], others experience a lack of mentoring and guidance [3], or are simply unaware of research activities at their university [13].

Similarly, undergraduate students might face similar barriers. In fact, some students might find that studying for their courses might be more promising than devoting time for medical research. In other cases, research experience does not meet their prior expectations in terms of mentoring or support, thus resulting in more perceived challenges. Within the AUB community, these barriers may be due to the progressive research oriented culture within AUB. In fact, the MRVP was only implemented in 2014 [6] and has yet to meet all students’ research related needs. Since its inception, the number of students matched on research projects has been growing. Moreover, students might lack guidance due to the nature of medical research at the undergraduate level, where some primary investigators might allocate less attention and importance to undergraduate students compared to that given to medical students or residents. A periodical follow up or meeting with the students’ mentors might help with this issue, where both parties would be more aware of their respective roles and responsibilities. We also speculate that many undergraduate students may not be aware of the research activities at their universities because they do not know its proper meaning and its prominence in their future careers, or might think that medical research distances them from clinical settings. Therefore, they might not monitor the research activities available at their universities. In order to help undergraduate students, universities can offer seminars about medical research at the beginning of the semester to educate them and avoid any misconceptions about the nature and importance of medical research.

### Limitations

The results of this paper should be viewed in light of its strengths and limitations. The main limitation of our study is the use of a non-validated questionnaire due to the absence of similar studies. Nevertheless, we have developed our questionnaire based on an extensive literature search, where a pilot test was also carried out as detailed in the methods above. Another limitation was the response rate (40%). One more point to consider is the non-response rate, which could have affected our results, although unlikely due to the random selection university-based sampling. A final limitation could be the generalizability of our results to other universities, although we believe students at AUB include a wide diversity of nationalities, backgrounds, and cultures.

## Conclusion

Our study has found that most students express a positive attitude, perception and willingness towards medical research, but mentoring is the most crucial element in ensuring ongoing positive experiences. Educators should focus on improving the undergraduate students’ medical research experience by enhancing the most prominent challenge faced by students: mentoring and guidance.

Many factors might influence the undergraduate students’ perception, attitude, and practice towards research, such as previous training, motivated faculty staff, and a rewarding environment. The assessment of undergraduate students’ perception, attitude, practice, and barriers towards medical research is thus crucial in boosting the overall medical research experience for undergraduate students. To our knowledge, this study is the first of its kind to assess these factors among undergraduate students.

## Data Availability

Please contact author for data requests.

## Abbreviations

MRVP: Medical Research Volunteer Program
AUB: American University of Beirut
AUBMC: American University of Beirut of Medical Center
FAS: Faculty of Arts and Sciences
FAFS: Faculty of Agricultural and Food Sciences
FHS: Faculty of Health Sciences
HSON: Rafic Hariri School of Nursing
GPA: Grade Percentage Average
